# Diseases of the Nose and Pharynx

**Published:** 1895-03

**Authors:** 


*Diseases of the Nose and Pharynx.* By JAMES B. BALL, M.D. Second Edition. Pp. xii., 364. London: BAILLIÈRE, TINDALL AND COX. 1894.

The present edition of this handbook has been considerably enlarged from the first edition, which treated of the nose and naso-pharynx only, while this includes the pharynx. This is an improvement, for it is absurd to separate diseases of the naso-pharynx from diseases of the pharynx. The book is eminently practical, for by avoiding theoretical discussions and by excluding references to, or descriptions of, illustrative cases, the author has kept it within a moderate compass and yet has included all that is of general interest.

There is perhaps no specialty—unless it be gynæcology—where the enthusiast is so apt to run riot as in rhinology, and we have great need to walk warily in order to avoid bringing discredit upon the whole subject. Dr. Ball speaks with the caution of the true man of science: he is no blind devotee; he treats of what he has seen and known, and in the main he may be safely followed.

We note with approval that the author recommends chloroform as the best anæsthetic for the removal of adenoids in young children, also that he recommends the patient to be lying on the right side with the head on a low pillow and bent a little forward. With this position and the use of Gottstein's curette there is nothing left to be desired. The chapter on diseases of the accessory sinuses is very complete: light is gradually being thrown on these terrible diseases, which so often lead to intracranial complications, and the author has kept himself abreast with the modern literature of the subject. In the treatment of abscess of the antrum, Dr. Ball recommends perforating through the socket of a tooth in preference to the method advocated by some of enlarging the natural aperture into the nose. We quite agree with him that the dependent opening is the best and gives the best results. The chapter on "Atrophic Rhinitis" is excellent, as also is that on "Hay Fever." In treating of deviations of the septum the author wisely advocates caution. Unless stenosis exists, giving rise to persistent catarrhal symptoms, interference is deprecated, but when operation is necessary the use of Bosworth's saw is advised. We fail to find anywhere in this work the wholesale removal of turbinated bones recommended, whether necrosing or varicosed, which is unfortunately so much in fashion at the present day; on the contrary, the extreme value of the presence of the turbinated bodies for healthy respiration is insisted upon, and we hope the day is far distant when the profession as a whole will endorse the barbarous method of treating slight affections of the nose by removal of unoffending necessary portions.

